# Analysis on charge-retention characteristics of sub-threshold synaptic IGZO thin-film transistors with defective gate oxides

**DOI:** 10.1038/s41598-024-62872-9

**Published:** 2024-05-24

**Authors:** Sungsik Lee

**Affiliations:** Department of Electronics Engineering, Pusan National University, Pusan, 46241 Republic of Korea

**Keywords:** Electrical and electronic engineering, Electronic devices

## Abstract

We provide a quantitative analysis on the charge-retention characteristics of sub-threshold operating In–Ga–Zn–O (IGZO) thin-film transistors (TFTs) with a defective gate-oxide for low-power synaptic applications. Here, a defective SiO_2_ is incorporated as the synaptic gate-oxide in the fabricated IGZO TFTs, where a defect is physically playing the role as an electron trap. With this synaptic TFT, positive programming pulses for the electron trapping are applied to the gate electrode, followed by monitoring the retention characteristics as a function of time. And this set of the programming and retention-monitoring experiments is repeated in several times for accumulating effects of pre-synaptic stimulations. Due to these accumulated stimulations, electrons are expected to be getting occupied within a deeper trap-state with a higher activation energy, which can lead to a longer retention. To verify these phenomena, a stretched exponential function and respective inverse Laplace transform are employed to precisely estimate a retention time and trap activation-energy for transient experimental results.

## Introduction

Synaptic devices, such as memristors^1–8^ and synaptic transistors^9–17^, have been intensively studied for neuromorphic applications. Among them, as an active-type synaptic device, field-effect transistors, such as thin-film transistors (TFTs), can be used as a synaptic transistor, where their synaptic functionalities, i.e. memory functionalities, are embedded in the gate insulator system. For example, a Ferro-electric dielectric or charge trapping layer (CTL) with a tunneling and blocking oxides can be incorporated into a gate insulator system in TFTs^18,19^. In particular, a CTL with a tunneling and blocking oxide layers is a typical structure to stably store electrons for a longer memory retention^19^. However, this type of gate insulator structures is sophisticated while requiring a relatively complicated fabrication-process, especially for a high quality gate-insulator stack. Also, a high programming pulse is required to get an electron trapping through the tunneling oxide. Interestingly, a TFT with a simple gate-insulator structure can also be used for a synaptic application^20,21^. Here, a single layer of the gate oxide should be defective, so electrons can be easily trapped at defects with a moderate positive-programming pulse^21^. In addition, when this synaptic TFT is operated in the sub-threshold region, a low-power synaptic performance can be achieved in comparison with the above-threshold operation^21^. However, a charge retention-time of this kind of devices seems to be much shorter compared to the typical synaptic TFT where a CTL is well defined between a tunneling and blocking oxides^19^. Here, it is unclear how its charge retention is improved with repeatedly applying multiple stimulations. Thus, the charge retention characteristics in the sub-threshold operating synaptic TFT with a defective gate-oxide is needed to be studied quantitatively.

In this paper, we present an analysis on the charge-retention characteristics of a synaptic TFT with a single layer of the gate oxide. For a low-power consumption, the synaptic TFT is operated in the sub-threshold region with a read voltage less than the threshold voltage. In the fabricated TFT, a defective SiO_2_ is used as the gate oxide while an amorphous In-Ga-Zn–O (IGZO) is employed as the channel layer. Here, a defect in the SiO_2_ is working as an electron trap. To the gate electrode, programming pulses for the electron trapping are applied. After that, the retention characteristics is monitored as a function of time. To accumulate effects of pre-synaptic stimulations, this sequential experiment, composed of the programming (P) and retention-monitoring (RM) processes (i.e. P-RM processes), is repeated in several times. After these repeated processes, it is found that electrons are being trapped with a higher activation energy, thus the electron trapping at deeper states. This eludes to a relatively long retention. To verify this, a quantitative analysis with a stretched exponential function (SEF) and inverse Laplace transform (ILT) is applied to respective experimental results while capturing a retention time and activation energy of traps.

## Results and discussion

### Synaptic depression with positive programming pulses

**Figure 1:**
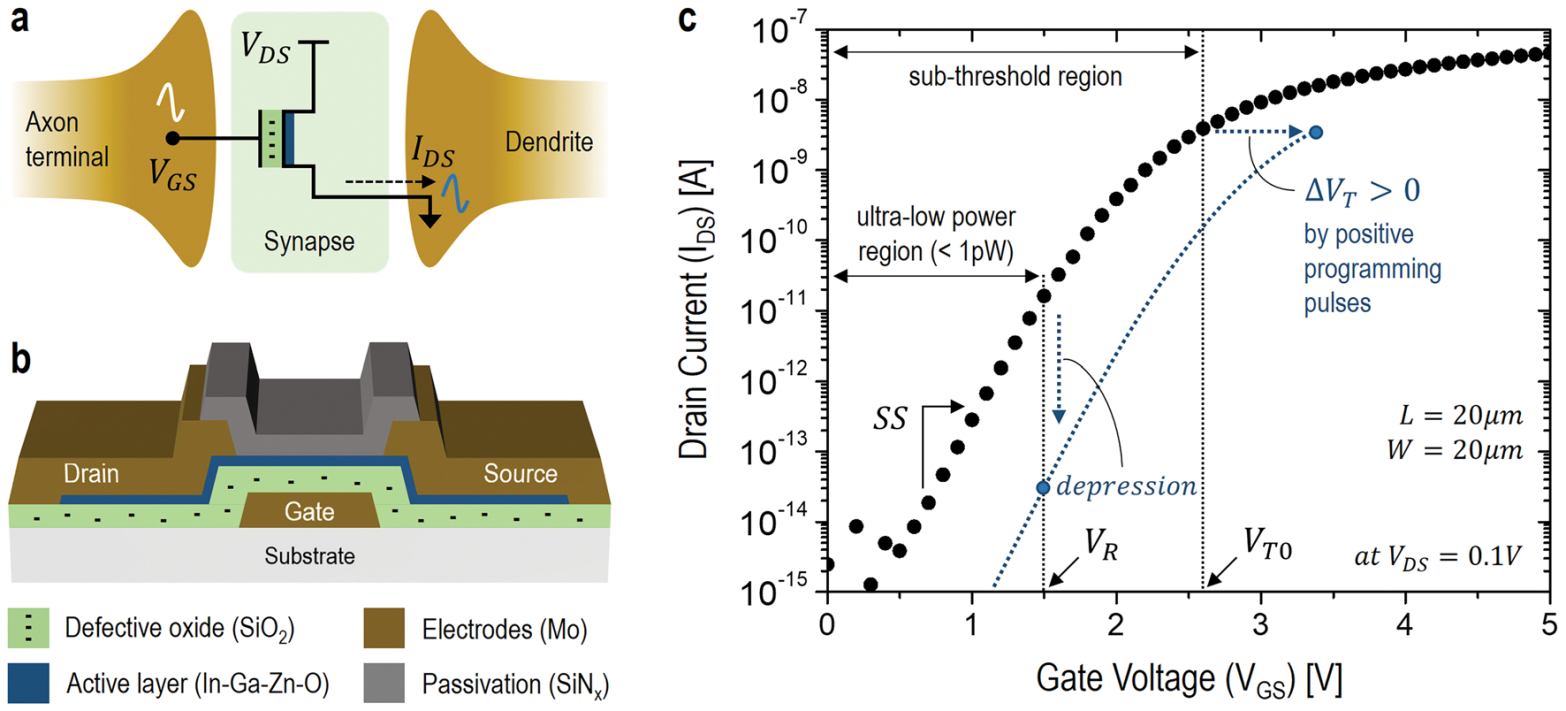
(**a**) Conceptual diagram of a synapse between a dendrite and axon terminal. (**b**) Schematic cross-sectional view of the fabricated synaptic TFT. (**c**) Measured transfer characteristics in the sub-threshold region of the synaptic TFT with the channel length (L) and width (W) of 20 µm, respectively.

Figure 1a shows a conceptual diagram of a synapse between a dendrite of the post-synaptic neuron and axon terminal of the pre-synaptic neuron. This biological structure can be mimicked with a synaptic thin-film-transistor (TFT). In the synaptic TFT, a gate insulator is used as an electron trapping layer for the synaptic functionality^21^. Also, the gate electrode is used for the axon terminal while the drain electrode is working as the dendrite. For representing a biological synaptic signal, the gate-source potential (V_GS_), i.e. a programming pulse, is applied as the pre-synaptic spike while the drain-source current (I_DS_) is monitored as the post-synaptic signal.

To realize these functionalities, an amorphous In-Ga-Zn–O (IGZO) TFT with a defective gate-oxide is prepared as the synaptic TFT. Here, the key for the synaptic functionality is the defective gate-oxide (SiO_2_) employed as the electron trapping layer, as can be seen in Fig. 1b^21^. As the key process for the synaptic functionality, after the gate electrode formation with Molybdenum (Mo), the defective SiO_2_ with 350 nm thickness is deposited with the plasma enhanced chemical vapor deposition (PECVD) at a low temperature, where the defects (e.g. trap states) can be created due to a low processing temperature. In this low-temperature PECVD process, the deposition temperature (T_d_) is set to 150 °C, which is 100 °C lower than a typical processing temperature (i.e. 250 °C), while applying other typical process conditions, such as the chamber pressure of 1 Torr and the source-gas flow ratio (N_2_O/SiH_4_) of 5. As the T_d_ is decreased, the defect density (D_t_) is expected to be increased, following the Arrhenius relation as D_t_ = D_t0_ exp(E_D_ / kT). Here, the D_t0_ is the reference defect density, E_D_ is an activation energy of defects, k is the Boltzmann’s constant, and T is the absolute temperature (i.e. T = 273 + T_d_). Based on this relation, when E_D_ is assumed to be 0.35 eV^22,23^, D_t_ in the SiO_2_ processed at T_d_ = 150 °C is estimated to be 10^14^ /cm^2^, which is an order of magnitude higher than D_t_ at T_d_ = 250 °C. If we assume the bigger value of E_D_, D_t_ can even be higher for the same amount of the decrease in T_d_. However, the higher D_t_ can lead to a lower dielectric-breakdown voltage as well as a higher leakage current^24,25^. To minimize this, the gate insulator is better to be thicker. So, this is one of reasons why a thick SiO_2_ of 350 nm is employed rather than a thin one. As another advantage of a thick gate insulator, the detrapping path for trapped electrons can be longer while minimizing electrons’ escape toward the gate electrode or channel layer. After this gate-insulator deposition, a 50 nm-thick IGZO for the channel layer is made with the RF-sputtering process, using an IGZO ceramic target, which subsequently patterned by the reactive ion etching (RIE)^10,21,26^. For the source and drain electrodes, Mo is deposited with a RF sputtering, and patterned with a wet etching. Finally, the device is passivated with a 200 nm-thick SiN_x_.

Figure 1c shows the measured transfer characteristics, i.e. I_DS_ vs. V_GS_, of the fabricated synaptic TFT for a low drain-source voltage (V_DS_) of 0.1 V. As can be seen, the initial threshold-voltage (V_T0_) and the sub-threshold slope (SS) are found to be about 2.6 V and 0.4 V/dec, respectively. For an ultra-low power synaptic operation, the sub-threshold region is chosen with the read voltage (V_R_) of 1.5 V less than V_T0_. Note that, as another benefit of the sub-threshold operation with a low V_DS_, the bias-induced electric-field can be minimized, leading to a less bias-stress and better reliability of the TFT. Also, for trapped electrons within the gate insulator, their drift toward the gate electrode or channel layer can also be reduced, thus a longer retention. Since the drain current (I_DS_) at V_R_ = 1.5 V is initially about 10pA for V_DS_ = 0.1 V, the maximum static power-consumption (P_max_) is about 1pW. As illustrated, a positive threshold-voltage shift (ΔV_T_ > 0) is expected by applying a positive programming pulse while I_DS_ at V_GS_ = V_R_ is to be decreased (i.e. depression).

**Figure 2:**
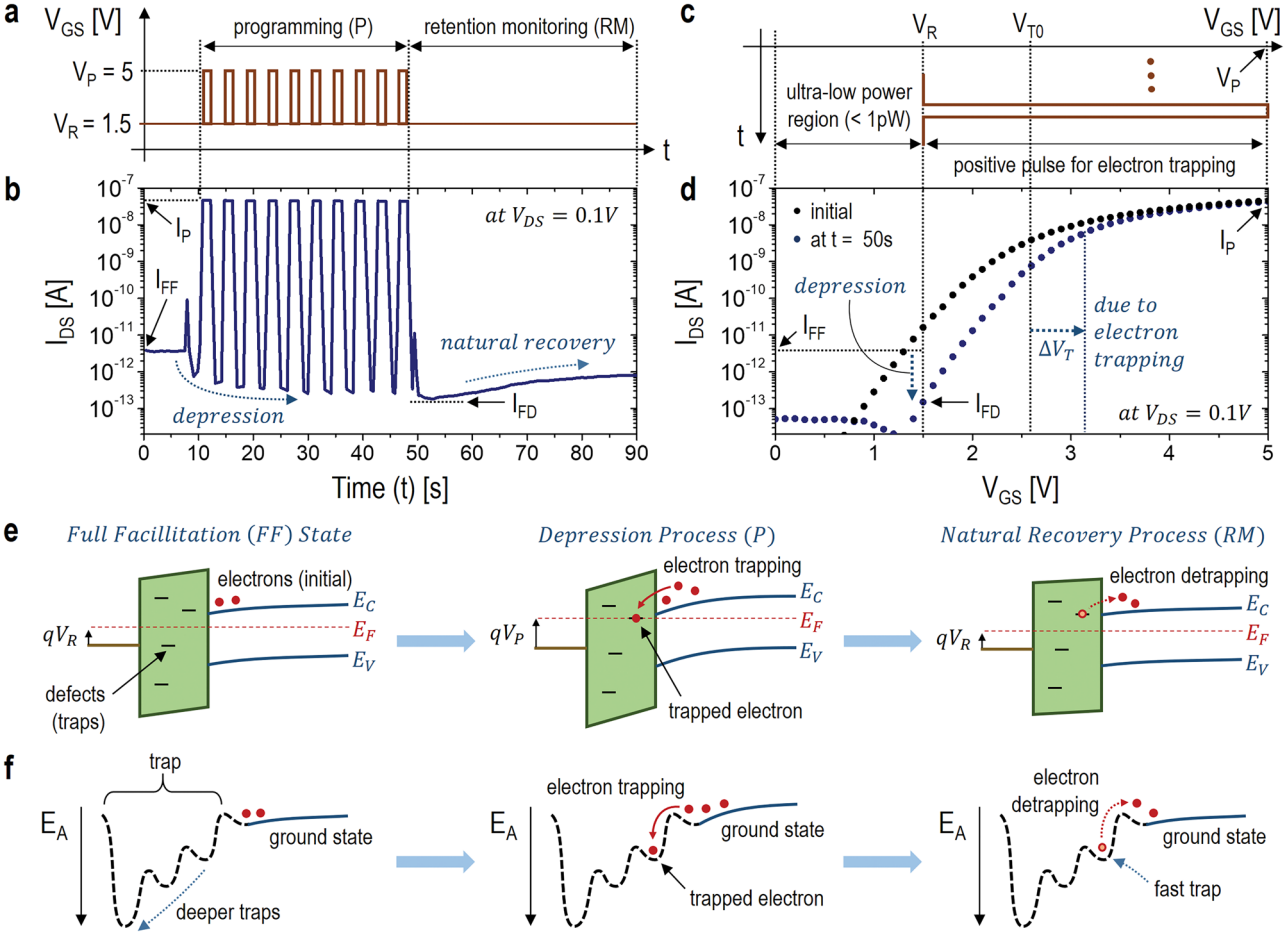
(**a**) Plot of programming pulses as a function of time. (**b**) Plot of I_DS_ upon the programming pulses as a function of time. (**c**) Plot of a single programming pulse as a function of time. (**d**) Transfer characteristics before and after applying programming pulses. (**e**) Band diagrams to describe electron trapping phenomena for the full facilitation, depression, and natural recovery processes, respectively. (**f**) Trap activation-energy diagrams to illustrate electron trapping phenomena for the full facilitation, depression, and natural recovery processes, respectively.

For an experimental observation of this I_DS_ depression, a programming (P) process is conducted with multiple programming pulses with the programming voltage (V_P_) of 5 V. And then, the retention monitoring (RM) process is followed, as shown in Fig. 2a. During the P and RM processes, I_DS_ for V_DS_ = 0.1 V is read at V_GS_ = V_R_ = 1.5 V. As seen in Fig. 2b, the initial I_DS_ at the full facilitation (FF), i.e. I_FF_, is about 3pA, which is decreased subsequently during the P-process. At t = 50 s, I_DS_ reaches about 0.1pA, which is a nearly full depression (FD), thus I_FD_ = 0.1pA. And its corresponding ΔV_T_ is about 0.6 V, as seen in Fig. 2c,d. Note that it is found that the SS after this P-process is almost maintained (see Fig. 2d), suggesting the current depression is due to the electron trapping rather than an interface quality degradation^27^. This current depression is due to the electron trapping into the defective gate-oxide. As illustrated in Fig. 2e,f, electrons are more likely to trapping into a fast trap-state near the ground state. For t > 50 s, the RM process is made. As can be seen in Fig. 2b, I_DS_ is slowly increased with a natural recovery process for V_GS_ = V_R_. This can be happen easily since the electron detrapping from the fast trap-state is easily made to the nearby conduction-band of the channel layer (i.e. ground state), as seen in Fig. 2e. Note that the ease of the electron detrapping out of the fast trap results in a short retention time. So, to extend the retention time, electrons should be trapped into a deeper trap-state (see Fig. 2f). To induce this phenomena, another P-process is followed right after the previous RM, suggesting the repeated P-RM processes.

### Memory characteristics with the repeated P-RM processes

**Figure 3:**
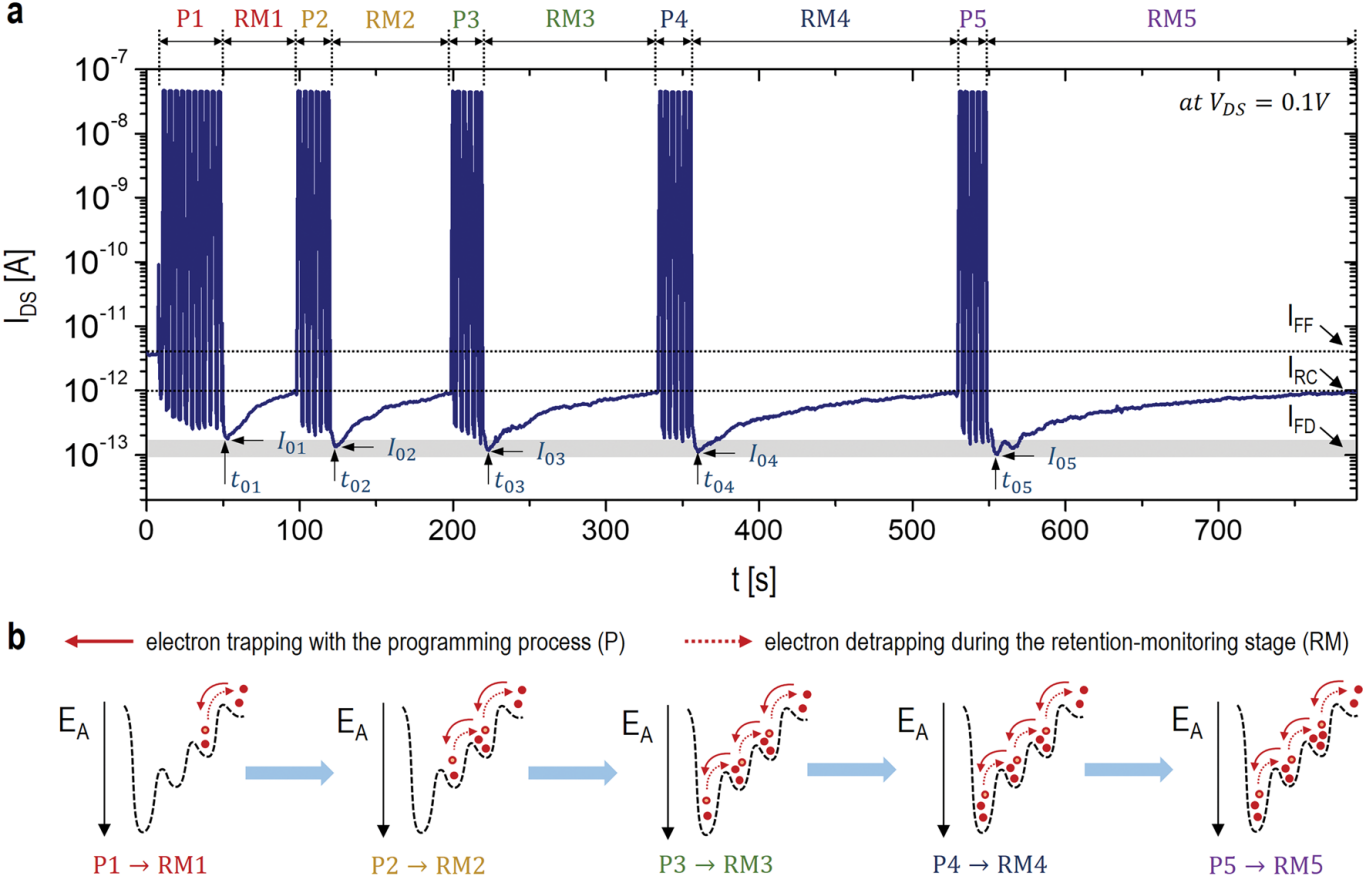
(**a**) Plot of I_DS_ with the repeated P-RM processes as a function of time. (**b**) Conceptual illustrations of the electron trapping and detrapping mechanisms which accumulated with the repeated P-RM processes.

To examine the effect of the repeated P-RM processes on the memory retention characteristic, 5 sets of the P and RM processes are sequentially applied to the fabricated synaptic TFT. Here, the P-process with a positive pulse is to get the electron trapping into the defective gate-oxide while the RM-process is followed to allow the electron detrapping into the channel layer. As seen in Fig. 3a, during the first P-process (P1) with 10 positive pulses, I_DS_ at V_R_ is depressed from I_FF_ of 3pA to I_FD_ of 0.1pA, which followed by the first RM-process (RM1). In this RM1 process, I_DS_ is slowly recovered from I_FD_ to I_RC_ of 1pA (i.e. recovery level), taking about 50 s of time. After that, the P2 is applied, where 6 positive pulses are applied to get the level of I_FD_ approximately. Once I_DS_ has reached I_FD_, the RM2 stage is processed until I_DS_ arrives at I_RC_. Here, we find that I_DS_ recovery to arrive at I_RC_ is found to be slower compared to the RM1 stage. This can be explained with some of electrons are being trapped into a deeper trap-state. In other words, some of the trapped electrons by the P1 are detrapped during the RM1, making the natural recovery. At the same time, some of the remaining electrons trapped at a fast state are moved further into a deeper state during the P2 process, making the retention extended, as described in Fig. 3b. This means that the synaptic stimulation is accumulated with the repeated P-RM processes while making the electron-retention longer (i.e. a longer-term memory). These effects can be accumulated more by applying more P-RM processes while satisfying I_FD_ = 0.1pA and I_RC_ = 1pA. As seen in Fig. 3a, this P-RM process is repeated by 5 times, eluding to the further extended retention-time proportional to the number of the P-RM processes. This implies that electrons within the defective gate-oxide are migrated toward deeper trap-states by the repeated P-RM processes (see Fig. 3b). To support this quantitatively, a relationship between a retention time and trap activation-energy is required to be analyzed while extracting their values.

### Analysis of the charge-retention characteristics with the SEF and ILT

In order to extract an accurate retention-time, while examining the recovery during each RM process, the stretched exponential function (SEF) is applied^27^. The general form of the SEF, i.e. F(t), is as follows,

1 $${\mathbf{F}}\left( {\mathbf{t}} \right) = {\mathbf{F}}_{0} \exp \left( { - \left( {\frac{{\varvec{t}}}{{{\varvec{\tau}}_{{{\varvec{eff}}}} }}} \right)^{{\varvec{\beta}}} } \right),$$

where F_0_ is the initial value at t = 0, τ_eff_ is an effective time-constant, and β is a stretched exponent. And F(t)/F_0,_ i.e. the normalized F(t), is defined as,

2 $${\overline{\mathbf{F}}}\left( {\mathbf{t}} \right) = \exp \left( { - \left( {\frac{{\varvec{t}}}{{{\varvec{\tau}}_{{{\varvec{eff}}}} }}} \right)^{{\varvec{\beta}}} } \right),$$

To apply Eq. 2, I_DS_ measured at the RM processes is normalized with the following equation^27^,

3 $$\overline{{{\mathbf{I}}_{{{\mathbf{DS}}}} }} \left( {\mathbf{t}} \right) = \frac{{\left[ {{\mathbf{I}}_{{{\mathbf{FF}}}} - {{\mathbf{I}}_{{{\mathbf{DS}}}} \left( {\mathbf{t}} \right) } } \right]}}{{{\mathbf{I}}_{{{\mathbf{FF}}}} - {\mathbf{I}}_{{{\mathbf{FD}}}}}}.$$

**Figure 4:**
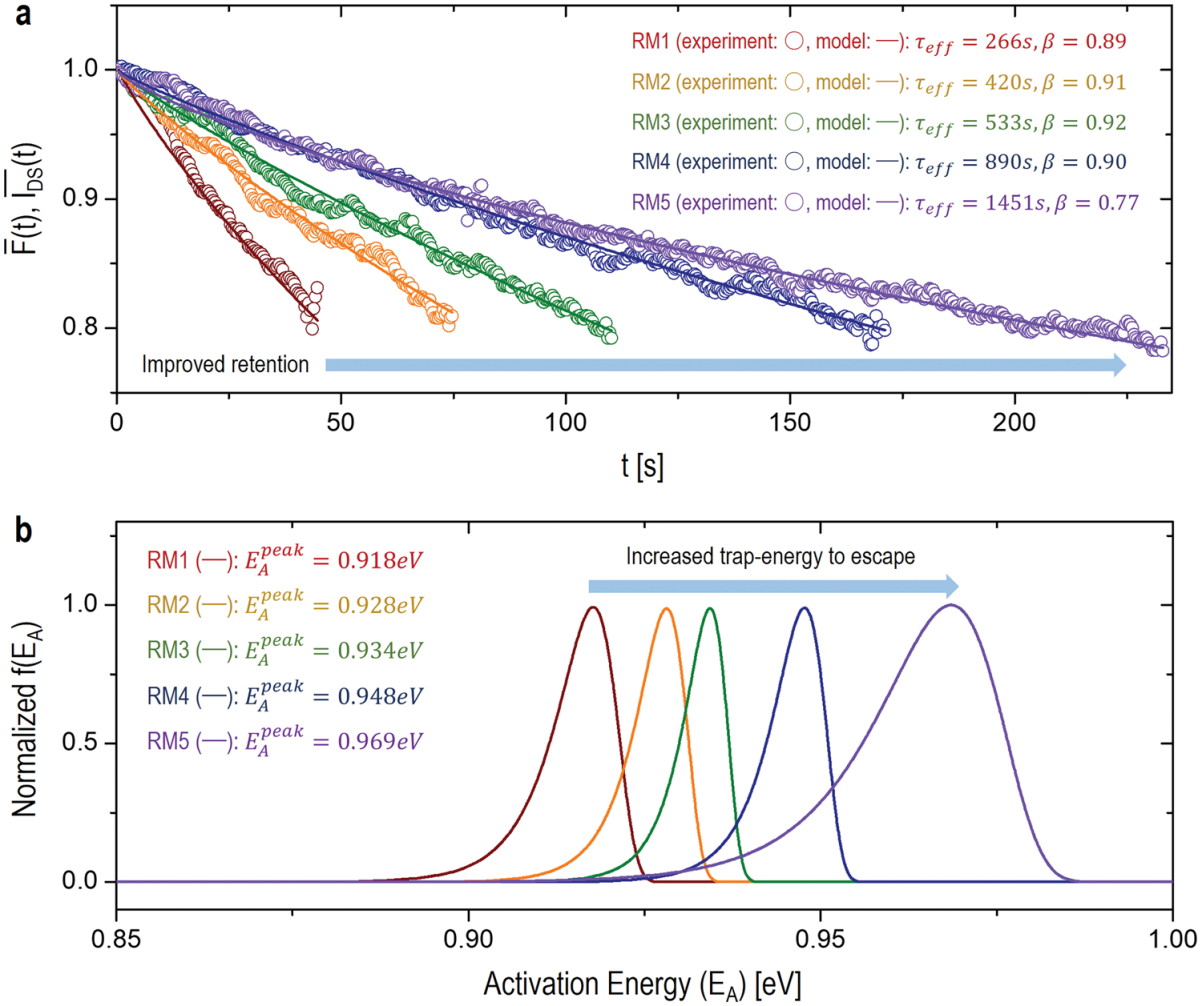
(**a**) Normalized F(t) for each RM process as a function of time, where the experiment results (i.e. normalized I_DS_(t)) are modelled with the SEF. (**b**) Normalized f(E_A_) for each RM process as a function of the activation energy (E_A_). Here, the extracted values of τ_eff_ and β for each case are applied to get the respective energy distribution.

Here, I_FF_ is 3pA, I_FD_ is about 0.1pA, and I_DS_(t) is the measured drain-current for the RM processes, as shown in Fig. 3a. With this Eq. 3, the experimental data is reconstructed (see Fig. 4a). While applying Eq. 2 to the reconstructed data for each RM process (RM1 ~ RM5), the values of τ_eff_ and β are retrieved for each case, respectively, as seen in the inset of Fig. 4a. From these results, as expected, it is found that τ_eff_ is gradually increased as the P-RM processes are repeated.

Based on these time-domain SEF results, we can also estimate the activation energy of traps for respective RM process. For this, it is needed to get an activation-energy distribution function (f(E_A_)). To get this function, as the first step, the frequency domain function (f(S)) is derived from the inverse Laplace transform (ILT) of $\overline{\mathbf{F}}\left(\mathbf{t}\right)$^27–29^, as,

4 $${\mathbf{L}}^{-1}\left\{\overline{\mathbf{F}}\left(\mathbf{t}\right)\right\}\equiv \mathbf{f}\left(\mathbf{S}\right)=\frac{1}{2{\varvec{\uppi}}\mathbf{j}}{\int }_{-\mathbf{j}\infty }^{\mathbf{j}\infty }\mathbf{exp}\left(-{\left(\frac{\mathbf{t}}{{{\varvec{\uptau}}}_{\mathbf{e}\mathbf{f}\mathbf{f}}}\right)}^{{\varvec{\upbeta}}}\right)\mathbf{exp}\left(\mathbf{S}\mathbf{t}\right)\mathbf{d}\mathbf{t}.$$

Here, S is a frequency. To convert this to an energy-distribution function, S is to be replaced with the relation of $\mathbf{S}={{\varvec{v}}}_{{\varvec{A}}{\varvec{E}}}\mathbf{exp}\left(-\frac{{{\varvec{E}}}_{{\varvec{A}}}}{{\varvec{k}}{\varvec{T}}}\right)$, where ${{\varvec{v}}}_{\mathbf{A}\mathbf{E}}$ is an attempt-to-escape frequency and kT is the thermal energy. Note that ${{\varvec{v}}}_{\mathbf{A}\mathbf{E}}$ sets to be 10^13^ /sec for this work^30^. Now, using a saddle point method, Eq. 4 is rewritten as the activation-energy distribution function, as follows,

5 $$\mathbf{f}\left({\mathbf{E}}_{\mathbf{A}}\right)=\frac{{{\varvec{\uptau}}}_{\mathbf{e}\mathbf{f}\mathbf{f}}{{\varvec{\beta}}}^{1+\frac{{\varvec{\gamma}}}{2}}}{\sqrt{2{\varvec{\pi}}{\varvec{\beta}}\left(1-{\varvec{\beta}}\right)}{\left({{\varvec{\tau}}}_{{\varvec{e}}{\varvec{f}}{\varvec{f}}}{{\varvec{v}}}_{{\varvec{A}}{\varvec{E}}}\mathbf{exp}\left(-\frac{{{\varvec{E}}}_{{\varvec{A}}}}{{\varvec{k}}{\varvec{T}}}\right)\right)}^{1+\frac{{\varvec{\gamma}}}{2}}}\mathbf{exp}\left(-\frac{\left(1-{\varvec{\beta}}\right){{\varvec{\beta}}}^{{\varvec{\gamma}}}}{{\left({{\varvec{\tau}}}_{{\varvec{e}}{\varvec{f}}{\varvec{f}}}{{\varvec{v}}}_{{\varvec{A}}{\varvec{E}}}\mathbf{exp}\left(-\frac{{{\varvec{E}}}_{{\varvec{A}}}}{{\varvec{k}}{\varvec{T}}}\right)\right)}^{{\varvec{\gamma}}}}\right),$$

where γ is defined as β/(1−β). Using the extracted values of τ_eff_ and β for each RM process, f(E_A_) can be drawn. As seen in Fig. 4b, the peak activation energy (${\mathbf{E}}_{\mathbf{A}}^{{\varvec{p}}{\varvec{e}}{\varvec{a}}{\varvec{k}}}$) of the normalized f(E_A_) is found to be increased as repeating the P-RM process. This indicates that electrons are being occupied at a deeper trap-state where an activation energy of traps is higher, thus a more difficulty for electrons to escape.

**Figure 5:**
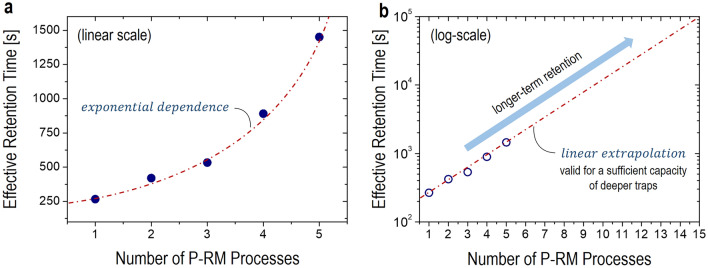
Effective retention time vs. number of P-RM processes in (**a**) linear scale and (**b**) log-scale, respectively.

Based on the results in Fig. 4, although the effect of the repeated P-RM processes are seems to be influentially accumulated making the electron trapping into a deeper trap-state of a higher activation energy, it can be argued that the retention time and activation energy are not dramatically increased. To clarify this, the dependence of τ_eff_ on the number of P-RM processes needs to be drawn. As seen in Fig. 5a, it is found that τ_eff_ has an exponential trend as increasing the number of P-RM processes. To verify this dependence, this plot is redrawn in a log-scale, showing clearly a linear dependence (see Fig. 5b). This also confirms its exponential dependence in the linear scale. By applying a linear extrapolation, as seen in Fig. 5b, it is estimated that τ_eff_ can be significantly extended up to 10^5^ s after 15 P-RM processes. Note that the estimated retention time for further P-RM processes can be limited depending on the capacity of deeper trap states. In addition, a multiple P-RM processes can lead to either an early insulator breakdown or increase of the gate leakage current. These results suggest that the synaptic TFT with a defective gate-oxide is possibly used for a longer-term retention although more P-RM processes are required assuming a presence of a sufficient capacity of deeper electron traps.

## Conclusion

In this article, we have shown a quantitative analysis on the charge-retention characteristics of sub-threshold operating In-Ga-Zn–O (IGZO) thin-film transistors (TFTs) with a defective gate-oxide for low-power synaptic applications. By applying the series of the P-RM processes to the fabricated synaptic TFT, it has been found that the electron retention time is exponentially increased as a function of the number of the P-RM processes, which has been quantitatively analyzed with the SEF. This implies the electron trapping into a deeper trap-state with a higher activation energy, which has been supported with the activation energy distribution deduced from the ILT of the SEF. Based on this analysis, it has been estimated that the retention time can be further extended up to 10^5^ s by applying 15 P-RM processes. These results have indicated that the synaptic TFT with a single defective gate-oxide can be enough for a longer-term memory although more P-RM processes are needed, which is valid for a presence of a sufficient capacity of deeper electron traps.

## Data availability

The datasets used and/or analysed during the current study available from the corresponding author on reasonable request.
